# *Csn3* Gene Is Regulated by All-*Trans* Retinoic Acid during Neural Differentiation in Mouse P19 Cells

**DOI:** 10.1371/journal.pone.0061938

**Published:** 2013-04-17

**Authors:** Rie Komori, Takanobu Kobayashi, Hikaru Matsuo, Katsuhito Kino, Hiroshi Miyazawa

**Affiliations:** Kagawa School of Pharmaceutical Sciences, Tokushima Bunri University, Sanuki, Kagawa, Japan; Baylor College of Medicine, United States Of America

## Abstract

κ-Casein (CSN3) is known to play an essential role in controlling the stability of the milk micelles. We found that the expression of *Csn3* was induced by all-*trans* retinoic acid (ATRA) during neural differentiation in P19 embryonal carcinoma cells from our study using DNA microarray. In this paper, we describe the detailed time course of *Csn3* expression and the induction mechanism of *Csn3* transcription activation in this process. The *Csn3* expression was induced rapidly and transiently within 24 h of ATRA treatment. Retinoic acid receptor (RAR)-specific agonists were used in expression analysis to identify the RAR subtype involved upregulation of *Csn3*; a RARα-specific agonist mimicked the effects of ATRA on induction of *Csn3* expression. Therefore, RARα may be the RAR subtype mediating the effects of ATRA on the induction of *Csn3* gene transcription in this differentiation-promoting process of P19 cells. We found that the promoter region of *Csn3* contained a typical consensus retinoic acid response element (RARE), and this RARE was necessary for ATRA-dependent transcriptional regulation. We confirmed that RARα bound to this RARE sequence in P19 cells. These findings indicated that the *Csn3* expression is upregulated via ATRA-bound RARα and binding of this receptor to the RARE in the *Csn3* promoter region. This will certainly serve as a first step forward unraveling the mysteries of induction of *Csn3* in the process of neural differentiation.

## Introduction

κ-Casein (CSN3) is the major protein component of milk micelles in most mammalian species. CSN3, which is mainly located at the surface of the micelles, is known to play an essential role in controlling the stability of the micelles [1], [2]. CSN3 is not only particularly important from the nutritional aspects, they are also known to have some other features. For example, previous studies showed that CSN3 from bovine milk possessed molecular chaperone activity and functioned to prevent precipitation of the target protein [3]. Chaperone activity is important for normal brain function and for neural cell differentiation [4]. Protein aggregation and misfolding are associated with many neurodegenerative diseases, including Alzheimer’s disease, and Parkinson’s disease. Several studies have shown that molecular chaperones act to prevent protein aggregation and play key roles in the prevention of such diseases [5].

We found that the expression of κ-casein gene (*Csn3*) was induced rapidly (within a few hours) during neural differentiation in P19 cells treated with all-*trans* retinoic acid (ATRA), an active metabolite of the vitamin A, from our study using DNA microarray. Previous studies also demonstrate that ATRA stimulates *Csn3* expression during the process of neural differentiation in P19 cells [6], [7], but it was not studied in more detail and the molecular mechanisms controlling this phenomenon remain uncharacterized. The physiological function of CSN3 in neural differentiation has yet to be defined. It is very interesting to elucidate the function of the milk protein CSN3 in the induction of neural differentiation. Therefore, we decided to investigate the relation between CSN3 and neural differentiation using P19 cells.

Pluripotent mouse P19 embryonal carcinoma (EC) cells were derived from a teratocarcinoma formed by transplantation of a C3H/He mouse embryo into a host mouse testis [8]. P19 cells can be induced to differentiate into cell types of three germ layers (ectoderm, endoderm, or mesoderm) when exposed to the appropriate inducer and culture conditions [9]–[12]. P19 cells have been used extensively as an *in vitro* model system for the study of molecular mechanisms involved in cellular differentiation and early embryonic development [13]; moreover, several genes that play important roles in mammalian differentiation have been identified using P19 cells [14]–[18]. When P19 cells are grown as aggregates and exposed to 1 µM of ATRA, they differentiate into neurons and glial cells that exhibit characteristic neural morphology and express proteins commonly found in central nervous system (CNS) neurons, such as neuron-specific class III β-tubulin (Tuj1), neuronal nuclei (NeuN) and neurofilament proteins [9], [11], [13], [19], [20].

It has also been well known that ATRA is an efficient inducer of neural differentiation in ES and EC cells [21], including mouse P19 cells. ATRA regulates target gene expression via binding to and activating a nuclear all-*trans* retinoic acid receptor (RAR); a RAR forms a heterodimer with a 9-*cis* retinoic acid (RA) receptor (retinoid X receptor; RXR) [22]–[25]. ATRA is a ligand only to RARs, but 9-*cis* RA is a ligand for both RARs and RXRs [26], [27]. These receptors function as nuclear ligand-activated transcriptional regulators. RAR/RXR heterodimers affect gene expression by binding to specific DNA sequences: retinoic acid response elements (RAREs) in the transcriptional regulatory regions of target genes [28], [29]. The RARE consensus sequence consists of a direct repeat (DR) element, 5′-PuG(G/T)(T/A)CA-3′, commonly separated by 1, 2 or 5 nucleotides (DR1, DR2 or DR5 motif, respectively) [24], [30]–[32]. In the absence of ligand, the heterodimeric receptor complexes interact with co-repressor proteins that prevent transcriptional activation of target genes [28]. When ATRA binds to RAR, the dissociation of the co-repressor is induced by the receptor conformational change, which allows the recruitment of co-activators to the complexes.

Here we note the fact that *Csn3* expression was induced within a few hours after induction of neural differentiation of P19 cells. The neural differentiation of P19 cells is induced by ATRA. To investigate whether *Csn3* was directly-regulated by ATRA in this process, we first analyzed the *Csn3* expression changes during the neural differentiation of P19 cells. Next, to elucidate the mechanism of regulation of *Csn3* expression by ATRA, we determined the RAR subtype and promoter region of *Csn3* involved in this regulation. The *Csn3* expression was induced rapidly and transiently within 24 h of ATRA treatment in P19 cells. This induction was regulated through RARα bound to a DR5 RARE in the 5′ flanking region of mouse *Csn3*. In addition, *Csn3* was also induced during neural differentiation by ATRA in mouse ES cells and developing mouse embryo. These results indicated that *Csn3* was controlled by ATRA in the process of neural differentiation. This study represents an initial step toward elucidating the function of *Csn3* in neural differentiation.

## Results

### *Csn3* was induced during neural differentiation in ATRA-treated mouse P19 cells

**Figure 1:**
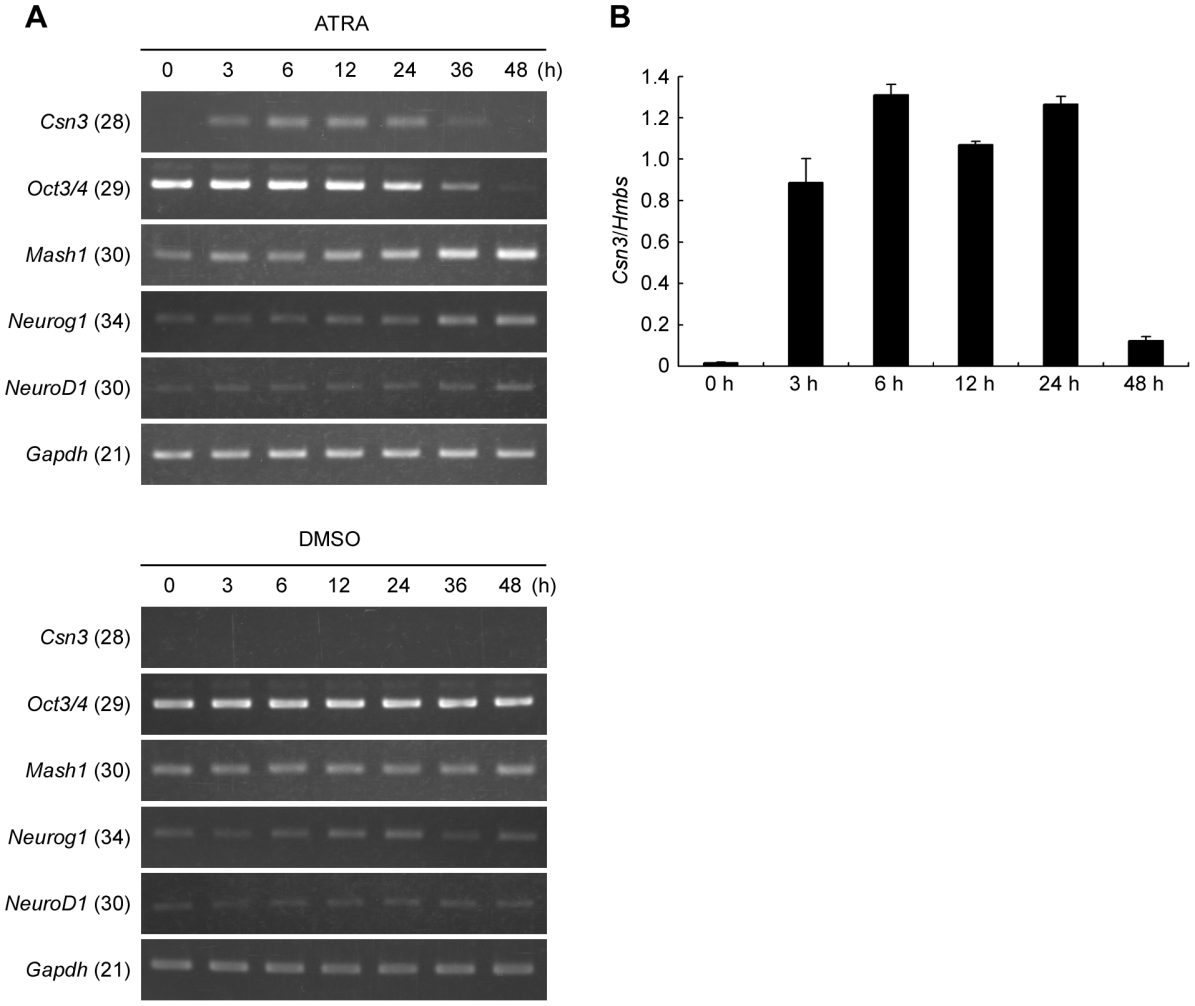
ATRA-dependent induction of *Csn3* gene expression in P19 cells. (**A**) Time course RT-PCR analysis of *Csn3* mRNA expression in P19 cells. Total RNA was extracted from P19 cells treated with ATRA (upper panel) or DMSO vehicle (lower panel) after 0, 3, 6, 12, 24, 36, or 48 h. RT-PCR was performed using gene-specific primers as described in Materials and Methods. Expression of pluripotency marker (*Oct3/4*) and neurogenic bHLH gene (*Mash1*, *Neurog1* and *NeuroD1*) were also examined to show the differentiating status. Glyceraldehyde-3-phosphate-dehydrogenase (*Gapdh*) was used as a loading control. PCR products were then subjected to electrophoresis through a 1.5% agarose gel and stained with ethidium bromide. Numbers in parentheses next to the gene symbols indicate the number of PCR cycles. RT-PCR experiments were repeated at least three times with similar results, and representative pictures are shown here. (**B**) Real-time PCR analysis of *Csn3* mRNA expression in P19 cells following ATRA treatment. Total RNA extracted from P19 cells that had been treated with ATRA for 0, 3, 6, 12, 24, or 48 h was used as template in this analysis. Real-time PCR was performed to assess *Csn3* expression; and *Csn3* expression was normalized relative to expression of the housekeeping gene hydroxymethylbilane synthase (*Hmbs*). All data points represent the mean plus S.E. from three independent experiments.

*Csn3* was identified as a gene whose expression was significantly altered when we performed DNA microarray experiments and compared mRNA levels of P19 cells treated with ATRA dissolved in dimethyl sulfoxide (DMSO) to those treated with vehicle-alone (DMSO) as control. To confirm the microarray results and clarify the detailed time course of the *Csn3* expression, total RNA was extracted from P19 cells after 0, 3, 6, 12, 24, 36, or 48 h of treatment with ATRA or DMSO and analyzed using RT-PCR and real-time PCR (Figure 1). To examine whether the conditions were set as appropriate for neural differentiation, we also monitored the expression of some neurogenic basic helix-loop-helix (bHLH) transcription factor genes such as *Mash1* (also known as *Ascl1*), *neurogenin1* (*Neurog1*), and *neurogenic differentiation 1* (*NeuroD1*) that promoted neuronal differentiation, and a pluripotency marker *Oct3/4* (also known as *Pou5f1*) (Figure 1A). As reported previously [33], [34], *Mash1* was upregulated, and *Oct3/4* was downregulated in ATRA-treated P19 cells, while the expression levels of these genes were not significantly changed in control cells. This observation demonstrated that ATRA-treated P19 cells lost pluripotency and effective neurogenesis occurred. *Neurog1* expression was slightly higher in ATRA-treated cells than in control cells after 36 and 48 h of treatment, while there was no significant difference in *NeuroD1* expression change between these two groups. Because the expression of these neuronal marker genes were known to be upregulated during days 3–5 of differentiation with ATRA [34], these results indicated that our approach of this paper analyzed the very early stage of neural differentiation in P19 cells.

Under such conditions, *Csn3* mRNA level was very low (undetectable by RT-PCR) immediately after treatment with ATRA (at 0 h); however, *Csn3* mRNA levels were induced within 3 h after the start of ATRA treatment, reached a plateau within 6 h, remained high for up to 24 h, and returned to the basal level by 48 h (Figure 1A, upper panel). In control cells in which neural differentiation was not induced, *Csn3* mRNA levels were undetectable via RT-PCR at each time-point (Figure 1A, lower panel). Results similar to those obtained using RT-PCR analysis were obtained using real-time PCR analysis (Figure 1B). These results indicated that the expression of *Csn3* was rapidly and transiently induced during neural differentiation in ATRA-treated P19 cells, suggesting that *Csn3* was regulated by ATRA during neural differentiation of P19 cells.

### ATRA-induced *Csn3* expression mediated through retinoic acid receptor α (RARα)

**Figure 2:**
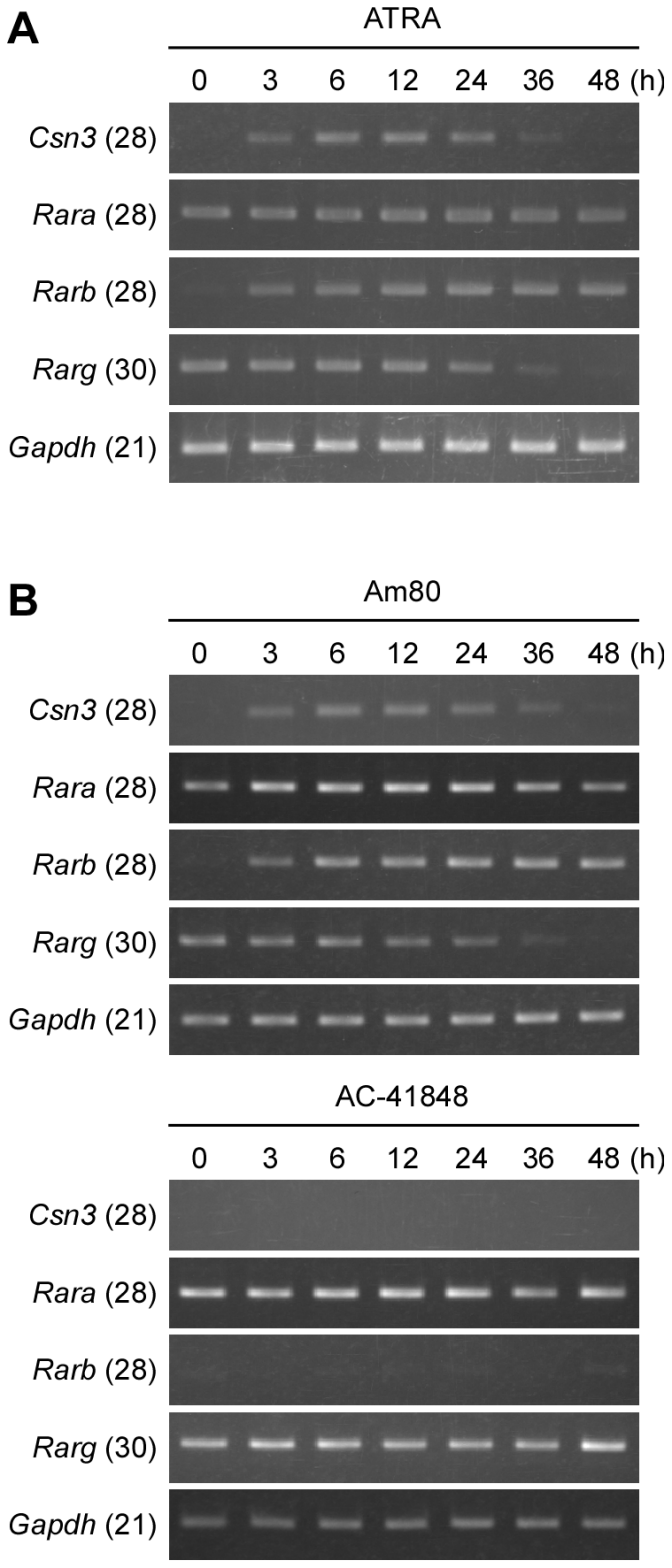
Effects of RAR agonists on *Csn3* expression in P19 cells. (**A**) RT-PCR analysis of the expression of *Csn3* and genes encoding the RAR subtypes (*Rara*, *Rarb*, and *Rarg*) during the neural differentiation in P19 cells. Total RNA was isolated from P19 cells at the time indicated following ATRA treatment, and used for cDNA synthesis. PCR analysis was performed with primer sets specific for *Csn3*, each *Rar* or for *Gapdh*. PCR products were then subjected to electrophoresis through a 1.5% agarose gel and subsequently stained with ethidium bromide. This experiment was repeated three times with similar results, and representative pictures are shown here. (**B**) RT-PCR analysis of *Csn3*, *Rara*, *Rarb*, and *Rarg* mRNA expression in P19 cells treated with RAR agonists. Total RNA was extracted from P19 cells treated with 100 nM Am80 (RARα agonist; upper panel) or 100 nM AC-41848 (RARγ agonist; lower panel), and *Csn3* and genes encoding the RAR subtypes expressions were evaluated by RT-PCR analysis. *Gapdh* was used as a loading control. Numbers in parentheses next to the gene symbols indicate the number of PCR cycles. RT-PCR experiments were repeated at least three times with similar results.

ATRA regulates target gene expression via binding to RAR [22]–[25]. The RAR family comprises three receptor subtypes: α, β, and γ [35]–[37]. To identify which RAR subtype was involved in the induction of *Csn3*, we analyzed the expression of each RAR gene (*Rara*, *Rarb* and *Rarg*) in P19 cells after ATRA treatment (Figure 2A). The change of gene expression of *Csn3* in ATRA-treated P19 cells was accurately reproduced as shown in Figure 1A. The *Rara* and *Rarg* mRNAs were detectable in P19 cells at 0 h of treatment. The level of *Rara* mRNA remained stable during the 48-h ATRA treatment, but *Rarg* mRNA levels decreased significantly following exposure to ATRA, and *Rarg* mRNA was almost undetectable at 48 h. On the other hand, *Rarb* mRNA was absent or undetectable at 0 h, significantly induced after 3 h of ATRA treatment, and remained constant until 48 h. This pattern of increase in *Rarb* mRNA was similar to that of *Csn3*, except that the expression of *Csn3* decreased after 24 h. These results indicated that either *Rara* or *Rarg*, but not *Rarb*, was involved in induction of *Csn3* expression.

Next, to determine which of the RAR subtypes, RARα or RARγ, was involved in the ATRA-dependent induction of *Csn3* expression in P19 cells, the effects of selective RAR agonists on induction of *Csn3* gene expression in P19 cells were analyzed. P19 cells were treated with a synthetic RARα-specific agonist, Am80 (Tamibarotene) [38], or a RARγ-specific agonist, AC-41848 [39], instead of ATRA, and the *Csn3* and each RAR gene expressions were assessed by RT-PCR (Figure 2B). The *Rara* mRNA levels remained nearly constant during 48 h in Am80- and AC-41848-treated cells. These patterns of *Rara* expression were similar to that in the ATRA-treated cells (Figure 2A vs. Figure 2B). There was a striking difference in expression of *Rarb* between Am80- and AC-41848-treated cells. In Am80-treated cells (Figure 2B, upper panel), *Rarb* mRNA was induced in the same manner as that in ATRA-treated cells. In contrast, *Rarb* mRNA levels were undetectable at each time-point in AC-41848-treated cells (Figure 2B, lower panel). These observations were consistent with previous findings that indicated the expression of RARβ was induced by ATRA via an RARα-dependent pathway [40]. A similar pattern of decreasing in *Rarg* mRNA was observed in ATRA- and Am80-treated cells, while the levels in AC-41848-treated cells remained stable during the 48 h of treatment. Under these conditions, Am80 caused a transient increase in *Csn3* expression that began within 6 h of the start of treatment and lasted through 24 h of treatment (Figure 2B, upper panel), but AC-41848 did not cause any increase in *Csn3* gene expression (Figure 2B, lower panel). These results indicated that ATRA regulated the expression of *Csn3* via RARα.

### A functional RARE is present within the promoter of the mouse *Csn3*

**Figure 3:**
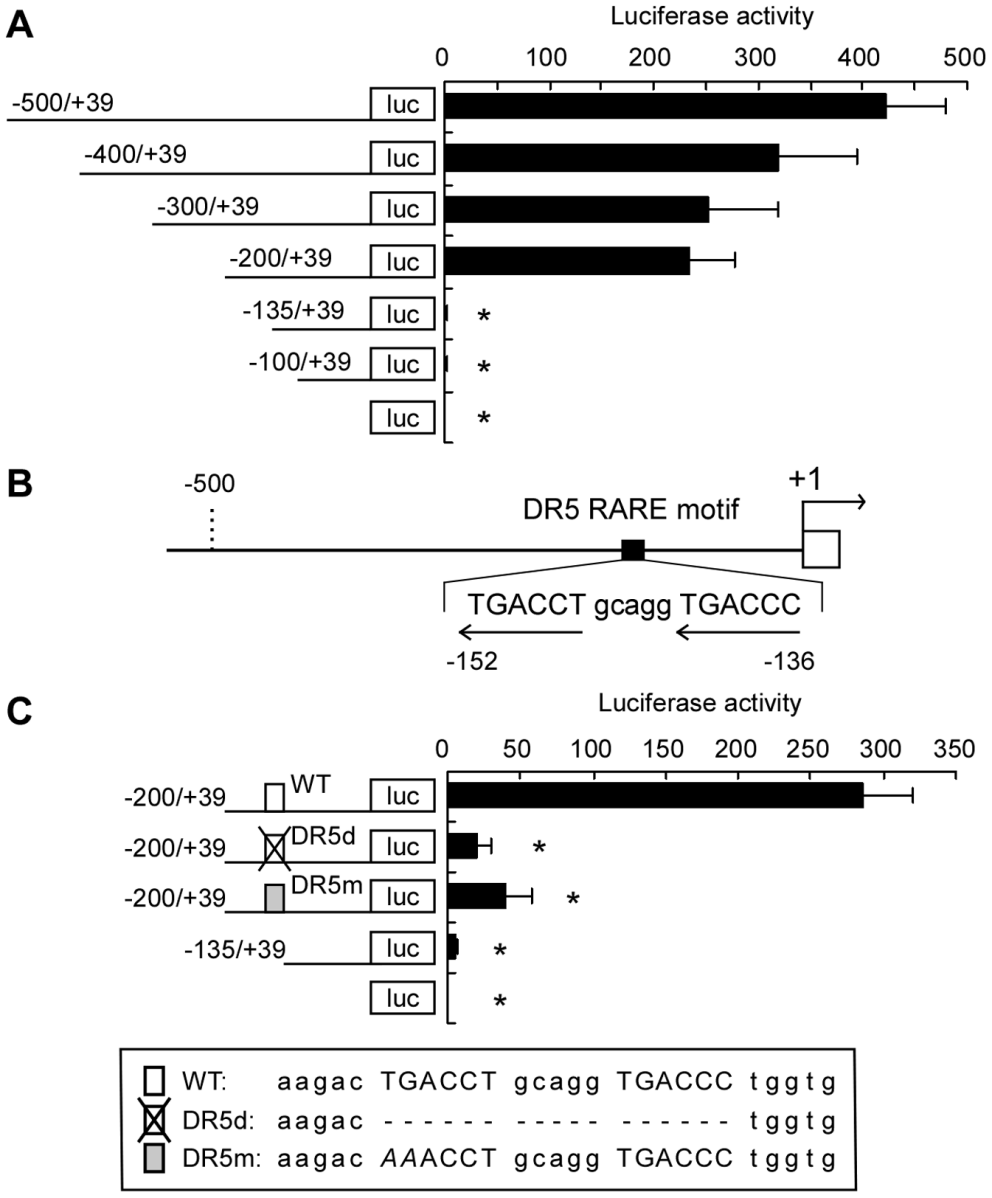
Identification of transcriptional regulatory region of the mouse *Csn3*. (**A**) Transcriptional regulatory activity of the *Csn3* proximal promoter in P19 cells. Schematic representations of the *Csn3* promoter 5′-deletion constructs used for transient transfections are shown on the left. P19 cells were transfected with the indicated deletion constructs and then treated with ATRA for 48 h followed by luciferase activity determination. Luciferase activity (right) was normalized against *Renilla* luciferase activity derived from cotransfected pGL4.74[*hRluc*/TK] reporter vector. Values represent the mean plus S.D. from three independent experiments. Student’s *t*-test was used to assess statistical significance. Asterisk indicates *P*<0.01 compared with value of construct containing the region between −500 to +39. (**B**) Map of the mouse *Csn3* promoter region. The putative DR5 RARE (filled box) and exon 1 (open box) are shown. The numbers indicate the positions relative to the transcriptional start site (+1). Arrows under the sequence indicate the location and orientation of the potential half sites for RAR/RXR binding of putative DR5 RARE (−152/−136). (**C**) Identification of functional sites within the mouse *Csn3* promoter region. P19 cells were transfected with wild-type (WT), deleted (DR5d), or mutated (DR5m) RARE-*luc* vector and treated with ATRA for 48 h. Schematic representations of the constructs and nucleotide sequence used for transfection are shown on the left and bottom panel, respectively. The mutated bases are indicated in italics. Luciferase activity (right) was normalized to *Renilla* luciferase activity. Values represent the mean plus S.D. from three independent experiments. Student’s *t*-test was used to assess statistical significance. Asterisk indicates *P*<0.01 compared with value of WT DR5 RARE-*luc* vector.

In most cases, the RAR/RXR heterodimer regulates gene expression by binding to the 5′ regulatory region of the target gene. We then used firefly luciferase reporter constructs and luciferase assays to examine the functionality of the *Csn3* promoter in P19 cells treated with ATRA; the reporter constructs represented a series of *Csn3* promoter deletion mutations in region from −500 bp upstream to +39 bp downstream of the transcriptional start site (Figure 3A). Each reporter construct was introduced into P19 cells, and luciferase activity was assayed following ATRA treatment. The constructs containing the sequences between −200 and +39 (−500/+39, −400/+39, −300/+39, and −200/+39) showed significant luciferase activity, whereas the constructs lacking the sequences between −200 and −136 (−135/+39, and −100/+39) showed little, if any, luciferase activity (Student’s *t*-test, *P*<0.01). These results indicated that the sequences from −200 to −136 were necessary for ATRA-dependent induction of *Csn3* promoter activity in P19 cells.

Analysis of the region between −200 and −136 of *Csn3* promoter revealed a putative DR5 RARE sequence beginning at −152 (TGACCTgcaggTGACCC; Figure 3B). To examine the importance of this sequence for ATRA-dependent luciferase activity in P19 cells, two constructs containing deleted or mutated *Csn3* DR5 RARE sequences (TGACCTgcaggTGACCC to *AA*ACCTgcaggTGACCC, nucleotides in italics were mutated as shown) were generated and used in luciferase assays (Figure 3C). The 17-bp deletion or the 2-bp mutation in the *Csn3* DR5 RARE (DR5d and DR5m constructs, respectively) resulted in significantly reduced luciferase activity when compared with the wild-type (WT) sequences (Student’s *t*-test, *P*<0.01). These results indicated that the −152/−136 sequence was a functional DR5 RARE, and it was required for ATRA-dependent induction of *Csn3* expression in P19 cells.

### RARα binds to the *Csn3* promoter containing the RARE site

**Figure 4:**
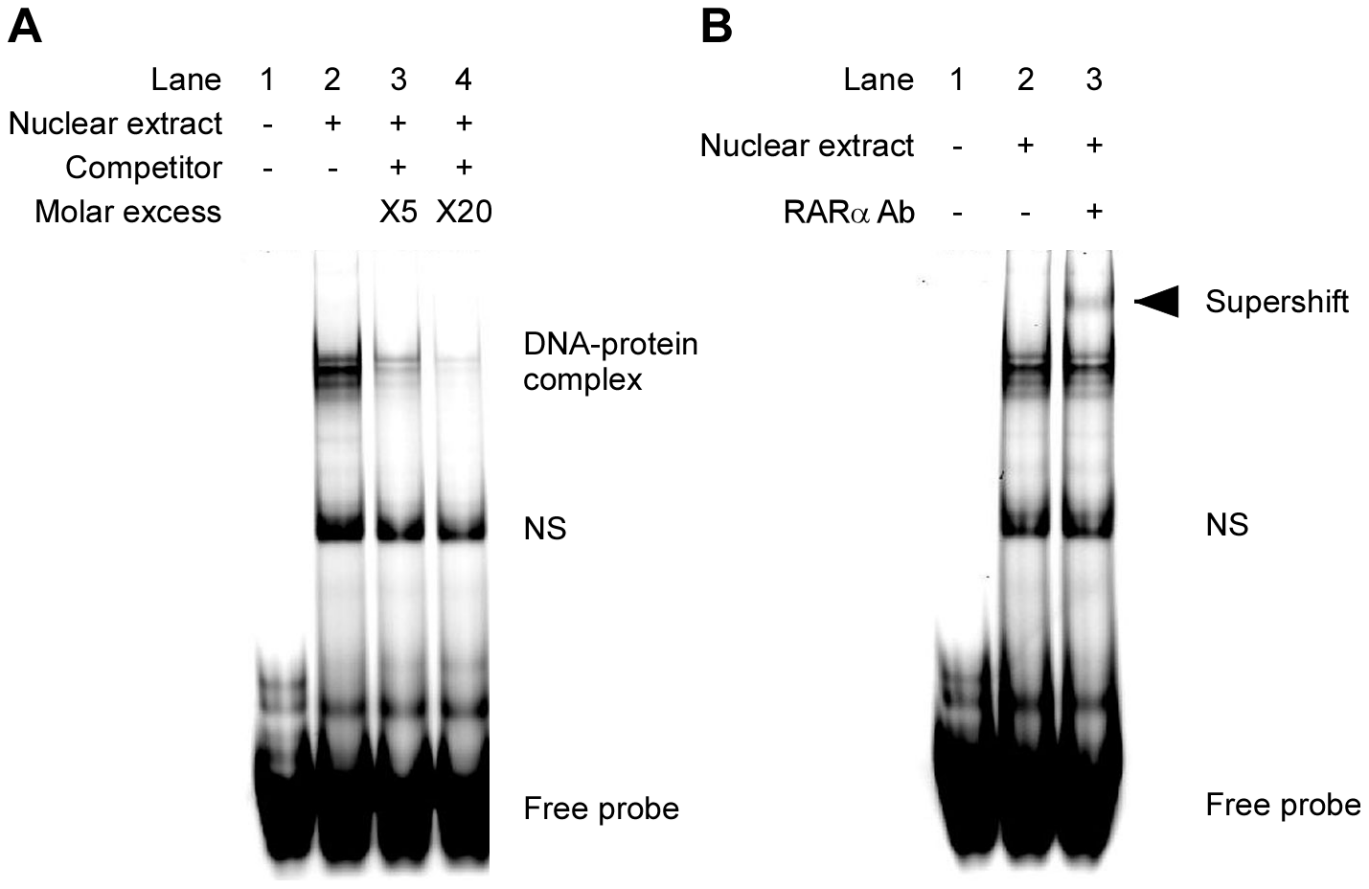
Molecular interaction between RARα and the *Csn3* promoter sequence containing the DR5 RARE motif. (**A**) Electrophoretic gel mobility shift assays (EMSAs) of the mouse *Csn3* DR5 RARE. EMSA was performed following incubation of Alexa680-labeled, double-stranded *Csn3* DR5 RARE probe corresponding to −160/−131 of *Csn3* promoter (5′-ACTAAGACTGACCTGCAGGTGACCCTGGTG-3′) with nuclear extract from P19 cells that had been treated with ATRA for 3 h (lane 2) or with no added protein (lane 1). For competition assay, unlabeled homologous oligonucleotides were added in increasing amounts (5- or 20-fold excess) to the binding reactions; the unlabeled oligonucleotides functioned as competitors with the labeled probe (lane 3 and 4, respectively). NS represents the non-specific interactions. (**B**) EMSA for assaying RARα binding to *Csn3* DR5 RARE. Alexa680-labeled *Csn3* DR5 RARE probe was incubated with no added protein (lane 1) or with nuclear extract from P19 cells that had been treated with ATRA for 3 h (lane 2). Supershift assay was performed by addition of anti-RARα antibody (lane 3). The arrowhead indicates the supershifted band.

It was found that DR5 RARE within the *Csn3* promoter was essential for the control of its transcription in P19 cells. Because our results indicated that RARα was involved in ATRA-dependent regulation of *Csn3* expression (Figure 2), we speculated that RARα bound to this DR5 RARE in P19 cells. In order to confirm this, we performed electrophoretic mobility shift assays (EMSAs) and analyzed the interaction between the DR5 RARE sequence in the *Csn3* promoter and nuclear proteins of P19 cells. Nuclear extracts were prepared from P19 cells that had been treated with ATRA for 3 h; the extracts were then incubated with Alexa680-labeled probe containing a centrally located the *Csn3* DR5 RARE element (5′-ACTAAGACTGACCTGCAGGTGACCCTGGTG-3′); these mixtures were then subjected to EMSA analysis (Figure 4A). The migration of the labeled DNA was retarded in a lane containing the DNA-extract mixtures (Figures 4A, lane 2). This observation indicated that DNA-protein complexes had formed in the presence of nuclear extract from P19 cells. To verify the specificity of these interactions, competition experiments were performed with excess amounts of unlabeled probe. It was competed by the unlabeled probe in a concentration-dependent manner (Figure 4A, lanes 3 and 4), so binding between labeled probes and nuclear proteins appeared to be sequence specific and the retarded band was indeed the probe containing *Csn3* DR5 RARE.

To determine whether RARα binds to the *Csn3* DR5 RARE sequence, anti-RARα antibody was used in an antibody supershift assay (Figure 4B). Nuclear extracts from P19 cells that had been treated with ATRA for 3 h were incubated with Alexa680-labeled probe in the presence of anti-RARα antibody. The retarded DNA-protein complexes were supershifted when incubated with antibody against RARα (Figure 4B, lane 3). These results indicated that RARα associated with the sequence between −160 and −131 of the *Csn3* promoter that contained the DR5 RARE motif and that this association was specific.

**Figure 5:**
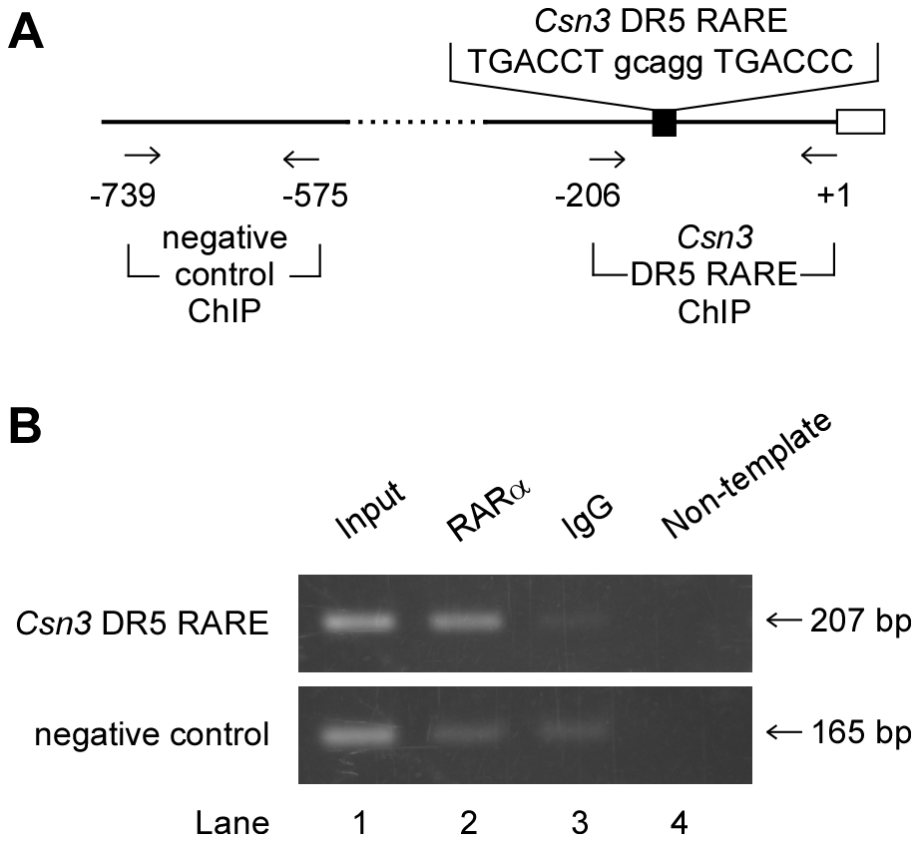
Binding of RARα to the *Csn3* promoter region containing the DR5 RARE motif. (**A**) Schematic illustration of the mouse *Csn3* promoter region. The *Csn3* DR5 RARE (filled box) and exon 1 (open box) are shown. Arrows under the nucleotide indicate the positions of the PCR primers used for chromatin immunoprecipitation (ChIP) assays. The numbers indicate position relative to the transcriptional start site (+1). (**B**) ChIP assays of RARα in *Csn3* promoter region in P19 cells treated with ATRA. DNA sequences within chromatin that had been immunoprecipitated with anti-RARα antibody (lane 2) or non-specific mouse IgG (negative control, lane 3) were amplified using primer pairs for the region including the DR5 RARE in *Csn3* promoter (−206 to +1) or the distal region from the target site (−739 to −575). Aliquots of the chromatin before immunoprecipitation were used as a positive control (Input, lane 1). Lane 4 contains non-template DNA.

Next, we used a ChIP assay to verify that RARα bound to the *Csn3* promoter in P19 cells (Figure 5). DNA-protein complexes were extracted from P19 cells that had been treated with ATRA for 3 h, and were immunoprecipitated with an anti-RARα antibody or a non-specific mouse IgG; DNA sequences in the immunoprecipitates were amplified using PCR and primer pairs specific for either a *Csn3* DR5 RARE site (−206 to +1) or a negative control site (−739 to −575) (Figure 5A). For each primer pair; a PCR product of the expected length was amplified from the Input samples (Figure 5B, lane 1). However, when *Csn3* DR5 RARE site were analyzed, the chromatin immunoprecipitated with the anti-RARα antibody generated a stronger PCR band (Figure 5B, lane 2) than did the chromatin precipitated with the non-specific IgG (Figure 5B, lane 3) (Figure 5B, upper panel), but very little PCR product was amplified when negative control site was analyzed (Figure 5B, lower panel). The control samples extracted from P19 cells that had been treated with DMSO vehicle for 3 h were also analyzed, but there were no significant differences between ATRA-treated and DMSO-treated groups (data not shown). These results indicated that the −206/+1 region containing the DR5 RARE of the *Csn3* promoter was physically occupied by RARα.

### *Csn3* was induced during neural differentiation in mouse ES cells and in developing mouse embryo

**Figure 6:**
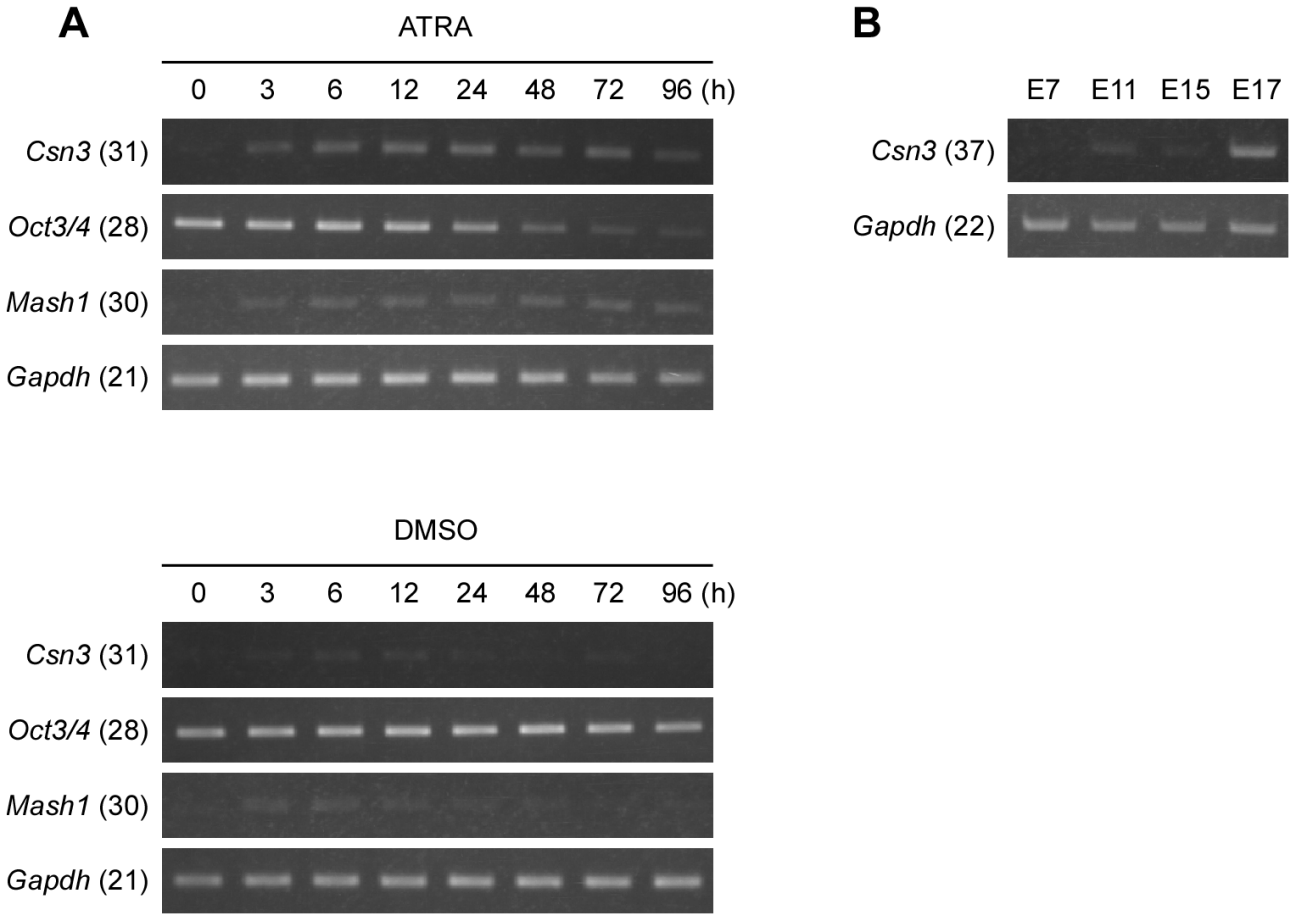
Expression of *Csn3* in mouse ES cells and in mouse embryonic development. (**A**) Time course RT-PCR analysis of *Csn3* mRNA expression in mouse ES cells (EB5). Total RNA was extracted from EB5 cells treated with ATRA (upper panel) or DMSO vehicle (lower panel) after 0, 3, 6, 12, 24, 48, 72 or 96 h. RT-PCR was performed using gene-specific primers as described in Materials and Methods. Expression of *Oct3/4* and *Mash1* were also examined to show the differentiating status. *Gapdh* was used as a loading control. PCR products were then subjected to electrophoresis through a 1.5% agarose gel and stained with ethidium bromide. Numbers in parentheses next to the gene symbols indicate the number of PCR cycles. (**B**) RT-PCR analysis of *Csn3* mRNA expression in mouse embryos at different developmental stages (E7–E17). The amplified PCR products from the mouse embryos were resolved in 1.5% agarose gel and stained with ethidium bromide. *Gapdh* was used as a loading control.

To investigate whether the effect of ATRA treatment on the induction of *Csn3* was limited to P19 cells, we used a mouse embryonic stem (ES) cell line (EB5). Our experiments revealed that *Csn3* expression was also upregulated by ATRA in mouse ES cells (Figure 6A). The expression profiles of marker genes indicated that ATRA induced neural differentiation in the ES cells. In addition, *Csn3* showed developmentally regulated expression during mouse embryogenesis (Figure 6B). These results indicated that the induction of *Csn3* in differentiation was a general phenomenon and was not restricted to the P19 cells.

## Discussion

From our study using DNA microarray, we found that the expression pattern of κ-casein gene (*Csn3*) was induced during the neural differentiation of mouse P19 cells. The *Csn3* expression was induced rapidly by ATRA in this process (Figure 1). The *Csn3* RNA level showed an increase with peak at 6 to 24 h after ATRA treatment, and this increase nearly disappeared within 48 h of the start of ATRA treatment. This finding is consistent with a previous study showing that the RNA of *Csn3* began to accumulate after 2 h of ATRA exposure in P19 cells [6]. In addition, we demonstrated that *Csn3* induction was quite transient and limited to the early stage of neural differentiation in mouse P19 cells.

It is well known that ATRA is able to increase their transcriptional activity through RAR that interacts with RARE in the promoter of the target gene. Alternatively, RAR can also affect transcription of some retinoid-responsive genes via interactions with other transcription factors in a RARE-independent mechanism. Recent studies on the regulation of gene expression by ATRA demonstrate that not all retinoid-responsive genes contain a RARE with the regulatory region [41]–[45]. These reports show that there are various mechanisms involved in the regulation of gene expression by ATRA, and then different mechanisms are used according to the target genes. Our results indicated that *Csn3* was a direct target of ATRA-activated RARα (Figure 2). Luciferase assay revealed that the *Csn3* promoter lost responsiveness to ATRA when sequences between −200 and −136 were deleted (Figure 3). A search of this region revealed that it contained a classical DR5 RARE and that this sequence was essential for ATRA-dependent induction of *Csn3* expression. With all these factors, we considered the possibility that this DR5 RARE motif may interact with RARα and *Csn3* induction can be regulated due to RARE-dependent transcription. As expected, EMSA and ChIP results indicated that this activated complex bound to a canonical DR5 RARE within the *Csn3* promoter (Figures 4, 5). Because RAR is a member of type II nuclear receptor, it is thought that RARα resides in the nucleus and binds to its response element regardless of whether ATRA (the RAR-specific ligand) is present or absent [46]. In this case, co-repressor and co-activator exchange is important for regulation of gene transcription. In fact, we confirmed that RARα bound to the *Csn3* DR5 RARE in P19 cells treated with DMSO vehicle using ChIP assay. Therefore, we concluded that the *Csn3* expression was regulated by ATRA-activated RARα through a common mechanism dependent on the DR5 RARE in P19 cells instead of interacting with other transcription factors binding to the different sites.

In this study, we show that the *Csn3* gene expression is induced by ATRA. Also some previous studies have demonstrated that several stimuli could induce upregulation of κ-casein gene expression. For example, *Csn3* expression was increased in a murine neuroblastoma cell line, SN56.B5.G4, following a low molecular weight β-amyloid (1–42) treatment [47] and in rat gastrocnemius muscle following botulinum toxin-A injection [48]. CSN3 protein is also reportedly a cellular prion protein (PrPC)-interacting protein based on a protein microarray analysis [49]. In any case, the κ-casein gene or protein was incidentally discovered from exhaustive expression analyses, and additional analyses about κ-casein were not conducted. Thus, the physiological significance of CSN3 protein or *Csn3* gene transcription in each process has not yet been revealed.

We investigated the regulation of *Csn3* gene expression using P19 murine EC cells. This cell line is commonly used as an *in vitro* model system for studying neurogenesis, because its critical developmental events closely resemble those of the early embryonic neuroectoderm. The neurons induced by culturing P19 cells exhibit many properties expected for CNS neurons. It has been reported that their morphology and expressed proteins were commonly found in CNS [9], [11], [13], [19], [20]. P19 cells express a variety of neurotransmitters and their cognate receptors [12]. They can develop into neurons with functional excitatory synapses and inhibitory synapses, and establish neuronal polarity [50].

Also, several reports have explored the possibility of cell transplantation therapy using P19 cells. P19 cells that have been implanted into the adult rat striatum survived and matured into functional neurons and glial cells within the transplantation site [51], [52]. When the neuronal progenitors derived from P19 cells were transplanted into the mouse cerebellum, they could settle in the host tissue and differentiate according to the surrounding conditions [53]. As P19 cells are produced from malignant teratocarcinoma, it is still arguable whether they are adequate source for cell replacement therapies in neurodegenerative disorders of the CNS. However, in either case, there is no doubt that this cell line is a highly suitable *in vitro* model system to study mammalian neurogenesis. Using such cell line, we found that the *Csn3* gene expression was upregulated through the RARα during neural differentiation. In addition, we confirmed that *Csn3* induction happened during the process of neural differentiation in mouse ES cells and mouse embryogenesis (Figure 6). From our results, we can speculate that CSN3 protein may play an important role in induction of differentiation and development.

As reported previously, the κ-casein null mouse strain have created and characterized [54]. According to their report, *Csn3*-deficient mice develop normally, while the females fail to lactate and suckle their pups. Their study was carried out to address the role of *Csn3* in lactation, and brain of *Csn3*-deficient mice has not been characterized. Therefore, it is not known the details about brain development and cranial nerve function of this strain. However, in view of the fact that no obvious phenotypic abnormality in the brain development was observed, *Csn3* may play a supplementary role in obtaining a smoother induction of neural differentiation.

There is a need to understand the possible biological significance of upregulation of *Csn3* during differentiation process. κ-Casein from bovine milk has been reported to possess some properties of molecular chaperones similar to those of intracellular small heat shock proteins (sHsps) and the extracellular protein clusterin [3]. Clusterin, which was initially identified as a secretory glycoprotein, was shown to possess a chaperone-like activity [55]. It has also been reported that clusterin interacts with particular protein and the interaction may act an important modulator during neuronal differentiation [56], and that clusterin can inhibit β-amyloid fibril formation by destabilization of pre-fibrillar species [57], [58]. Taken together, we thought it possible that CSN3 could act as a modulator, such as molecular chaperone, and provide a useful mechanism for more effective induction of neural differentiation in P19 cells. CSN3 may act to prevent aggregation of some kind of signal molecule required to trigger neural differentiation, and enable the molecule to fulfill its role. To this end, *Csn3* may be induced temporarily at a very early stage of neural differentiation, where it can facilitate the initiation of neural differentiation. Further investigations of the physiological functions of mouse CSN3 in promoting or facilitating neural differentiation are in progress.

## Materials and Methods

### Cell culture and neural differentiation

P19C6, a subclone of the P19 mouse EC cell line, and EB5, a mouse ES cell line, were used.

P19C6 was obtained from RIKEN BioResource Center through the National Bio-Resource Project of MEXT, Japan. P19 cells were cultured in α-minimum essential medium (α-MEM; Sigma), which was supplemented with 10% heat-inactivated fetal bovine serum (FBS; Gibco), 2 mM L-glutamine (Kanto Chemical), 100 units/ml penicillin (Nacalai Tesque), and 100 mg/ml streptomycin (Sigma). The cells were maintained at 37°C in an incubator infused with 5% CO_2_.

To induce neural differentiation, cells were aggregated in bacterial-grade dishes (Iwaki) at a seeding density of 2×10^5^ cells/ml in the presence of 1 µM of ATRA (Sigma) dissolved in DMSO (Sigma). Cell aggregates were collected 2 days after seeding, reseeded onto bacterial-grade dishes, and cultured with ATRA for 2 more days. The aggregates were then dispersed using a 0.25% trypsin (Sigma)-EDTA solution and reseeded onto tissue-culture-grade dishes coated with poly-D-lysine in a N2 serum-free medium (DMEM/F12 (Sigma) supplemented with 5 µg/ml insulin, 50 µg/ml human transferrin, 20 nM progesterone, 60 µM putrescine, and 30 nM sodium selenite); this medium also contained 1 µg/ml fibronectin (Gibco). The cells were then allowed to adhere to the culture dish and cultured medium was replaced every 2 days.

EB5 cells (a kind gift from Dr. Hitoshi Niwa, RIKEN Center for Developmental Biology, Kobe, Japan), a subline from mouse E14tg2a ES cells, carry the blasticidin S-resistant selection marker gene driven by the *Oct3/4* promoter, which is active in the undifferentiated state [59]. Undifferentiated EB5 cells were maintained on gelatin-coated (0.1%) dishes in Glasgow minimum essential medium (G-MEM; Sigma), which was supplemented with 10% heat-inactivated FBS (Gibco), 1× MEM Non-Essential Amino Acids (NEAA; Gibco), 1 mM sodium pyruvate (Gibco), 0.1 mM 2-mercaptoethanol (Wako Pure Chemical Industries), 1,000 U/ml ESGRO (Chemicon), and 10 µg/ml blasticidin S (Funakoshi). For differentiation, EB5 was aggregated in bacterial-grade dishes (Iwaki) in the presence of 1 µM of ATRA (Sigma) dissolved in DMSO (Sigma). Cell aggregates were collected 2 days after seeding, reseeded onto bacterial-grade dishes, and cultured with ATRA for 2 more days. The aggregates were then reseeded onto tissue-culture-grade dishes coated with poly-D-lysine as described above.

To identify the RAR subtypes, 100 nM of Am80 (RARα agonist, Sigma) or 100 nM of AC-41848 (RARγ agonist, Sigma) were used in place of ATRA.

### Reverse transcription-polymerase chain reaction (RT-PCR) and real-time PCR

Total RNA was isolated from P19 or EB5 cell pellets using the RNeasy Mini Kit (Qiagen). For RT-PCR analysis, cDNA was synthesized from a 1-µg sample of each total RNA preparation; random primers, SuperScript III reverse transcriptase (Invitrogen), and a PCR Thermal Cycler (Takara) were used according to the manufacturers’ instructions. The subsequent PCR amplification conditions were as follows: step 1, initial denaturation at 95°C for 10 min; step 2, amplification for 21–34 cycles of 95°C for 30 sec, 62°C for 30 sec, and 72°C for 1 min; and step 3, a final extension at 72°C for 7 min. PCR amplifications were performed using AmpliTaq Gold 360 Master Mix (Applied Biosystems) and gene-specific primers. The numbers of PCR cycle for each gene are described in Figures 1, 2 and 6. The primer sequences and the expected size of the amplified fragment for each PCR were as follows: *Csn3*, 5′-CAAACCCTACTGCCAAGCAAG-3′ and 5′-TTGTAGGCATGGCAAGAAAGG-3′, amplifying a 414-bp product; *Oct3/4*, 5′-GTTGGAGAAGGTGGAACCAAC-3′ and 5′-GGACTGAGTAGAGTGTGGTGA-3′, amplifying a 635-bp product; *Mash1*, 5′-CAACCGGGTCAAGTTGGTCAA-3′ and 5′-CCAGTTGGTAAAGTCCAGCAG-3′, amplifying a 322-bp product; *Neurog1*, 5′-CCTTTGGAGACCTGCATCTCT-3′ and 5′-CAGGGCCCAGATGTAGTTGTA-3′, amplifying a 426-bp product; *NeuroD1*, 5′-CTGATCTGGTCTCCTTCGTAC-3′ and 5′-GCACTCATGACTCGCTCATGA-3′, amplifying a 559-bp product; *Rara*, 5′-CGACGAAGCATCCAGAAGAAC-3′ and 5′-CGCAGAATCAGGATATCCAGG-3′, amplifying a 479-bp product; *Rarb*, 5′-AAGCCTGCCTCAGTGGATTCA-3′ and 5′-GCGCTGGAATTCGTGGTGTAT-3′, amplifying a 520-bp product; *Rarg*, 5′-GGAAGCTGTAAGGAACGATCG-3′ and 5′-TCCATTCGGTCTCCACAGATG-3′, amplifying a 558-bp product; and *Gapdh*, 5′-ACCACAGTCCATGCCATCAC-3′ and 5′-TCCACCACCCTGTTGCTGTA-3′, amplifying a 452-bp product. *Gapdh* served as a loading control to allow comparison of RNA levels among different samples. The PCR products were analyzed by electrophoresis in 1.5% agarose gels. The gels were stained with ethidium bromide and visualized by UV illumination. Each experiment was performed three times to confirm reproducibility. For expression analysis of *Csn3* during mouse embryogenesis, the normalized cDNA panels from four different developmental stages (E7, E11, E15, and E17 mouse embryos) included in MTC (Multiple Tissue cDNA) cDNA Panels (Clontech Laboratories) were used as templates.

For real-time PCR analysis, cDNA was synthesized from a 1-µg sample of each total RNA using random primers, a High Capacity cDNA Reverse Transcription Kit (Applied Biosystems), and a PCR Thermal Cycler (Takara) according to the manufacturers’ instructions. Gene-specific primers, cDNA template, and SYBR Green PCR Master Mix (Applied Biosystems) were used to PCR amplify target genes; amplification was run for 40 cycles using the 7500 Real-Time PCR System (Applied Biosystems). The instrument’s dissociation protocol was used to verify that only the specified PCR products were detected. The relative transcript amounts of *Csn3* were calculated using a standard curve generated with serial cDNA dilutions and normalized to that of *Hmbs* within the same cDNA sample. The primer sequences and the expected size of the amplified fragments from each PCR were as follows: *Csn3*, 5′-GGCATTAACTCTGCCCTTTTTG-3′ and 5′-TCACCACGGCAGTTTGAATC-3′, amplifying a 63-bp product; *Hmbs*, 5′-ACTCTGCTTCGCTGCATTG-3′ and 5′-AGTTGCCCATCTTTCATCACTG-3′, amplifying a 101-bp product.

### Plasmid construction

The DNA sequence of the *Csn3* 5′-flanking region (−500 to +39 where +1 represents the transcriptional start site of *Csn3*) was amplified from genomic DNA isolated from P19 cells. KOD-Plus DNA polymerase (Toyobo) and *Csn3* primers, 5′-GAGACTCGAGTTAAGACTGCTGATTTTTATT-3′ and 5′-GAGAAAGCTTTGGAGTCAATTCTTGCTTGGCAGT-3′, were used to amplify a PCR product; this product was then inserted into the multiple cloning site (XhoI and HindIII sites) of a luciferase reporter vector pGL4.10[*luc2*] (Promega) to make the pGL4.10/Csn3(−500/+39) construct. A series of 5′-truncated constructs were generated by PCR cloning using pGL4.10/Csn3(−500/+39) as the template with the following forward primers: −400/+39, 5′-GAGACTCGAGGTTCTTCAAGTAAAAACACTT-3′; −300/+39, 5′-GAGACTCGAGAGAGGTTAAGAATGCAATTAG-3′; −200/+39, 5′-GAGACTCGAGTGCTTATGACTCACATCTGTT-3′; −135/+39, 5′-GAGACTCGAGTGGTGACCTCAAATCTTGCCT-3′; and −100/+39, 5′-GAGACTCGAGTATTGGGTGAAAAGTAGGAGG-3′. The putative *Csn3* DR5 RARE (−152 to −136) was deleted or mutated (TGACCTGCAGGTGACCC to *AA* ACCTGCAGGTGACCC, mutation sites are in italics) using PCR-based mutagenesis. All of the primers contained XhoI or HindIII restriction enzyme site (underlined) to facilitate cloning. The sequence of each construct was verified by DNA sequencing using the BigDye Terminator version 3.1 Cycle Sequencing Kit on 3130/3130xl Genetic Analyzer (Applied Biosystems).

### Luciferase reporter assay

For luciferase assays, P19 cells were seeded into 24-well culture plates at a density of 2 × 10^4^ cells/well. Cells were incubated for 24 h and then subjected to transient transfection with the firefly luciferase reporter plasmid pGL4.10[*luc2*] (720 ng/well), which encoded a fragment of the *Csn3* promoter, and the pGL4.74[*hRluc*/TK] (80 ng/well) plasmid (Promega), which encoded *Renilla* luciferase as an internal control; Lipofectamine 2000 reagent (Invitrogen) was used for the transfections, and transfected cells were incubated for an additional 24 h. The transfected cells were then reseeded into 24-well plates for suspension culture (Sumilon) and treated with 1 µM of ATRA. After 48 h of incubation in ATRA, cells were harvested and luciferase activities were determined using the Dual-Glo Luciferase Assay System (Promega) according to the manufacturer’s instructions. Luminescence of luciferase was measured in a 96-well plate luminometer Mithras LB 940 (Berthold technologies). For each sample of cell extract, firefly luciferase luminescence was normalized to *Renilla* luciferase luminescence.

### Preparation of nuclear proteins and electrophoretic mobility shift assay (EMSA)

Nuclear extracts were prepared as described previously [60]. Briefly, P19 cells treated with 1 µM of ATRA for 3 h were subjected to centrifugation to form a cell pellet, and the cell pellet was then resuspended in hypotonic buffer (10 mM HEPES (pH 7.9), 10 mM KCl, 1.5 mM MgCl_2_, 0.5 mM DTT, Complete Mini EDTA-free protease inhibitors (Roche)) and incubated on ice for 10 min to disrupt the cells. Nuclei were collected from cell lysates by centrifugation at 1,000 *g* for 5 min and were then resuspended in hypertonic buffer (20 mM HEPES (pH 7.9), 420 mM NaCl, 1.5 mM MgCl_2_, 0.2 mM EDTA, 25% glycerol, 0.5 mM DTT, Complete Mini EDTA-free protease inhibitors (Roche)) and incubated on ice for 30 min. The supernatant, which contained nuclear proteins, was collected by centrifugation at 20,000 *g* for 2 min and stored at −80°C. All centrifugations were performed at 4°C. The protein concentration in each sample of nuclear extract was measured using the Bio-Rad Protein Assay Dye Reagent Concentrate (Bio-Rad); bovine serum albumin was used as a standard for the protein assays.

For EMSA, an Alexa680-labeled single-stranded oligonucleotide containing the putative *Csn3* DR5 RARE (5′-ACTAAGACTGACCTGCAGGTGACCCTGGTG-3′; the sequence corresponding to −160/−131 of 5′-region of *Csn3*) was annealed with an unlabeled complementary nucleotide and then used as a probe. Nuclear extracts (10 µg) were preincubated for 10 min on ice in reaction buffer containing 10 mM Tris-HCl (pH 7.5), 50 mM NaCl, 0.5 mM EDTA, 0.5 mM DTT, 1 mM MgCl_2_, and 2 µg poly(dI-dC)·poly(dI-dC). After incubation, the Alexa680-labeled double-stranded DNA probe was added to the preincubated extract and this mixture was incubated for 30 min at room temperature in a total volume of 20 µl. Binding reactions were separated by electrophoresis on 5% nondenaturing polyacrylamide gels (the ratio of acrylamide/bisacrylamide  =  29∶1) using 0.5× Tris-borate-EDTA buffer as running buffer for 165 min at 4°C, 170 V. After electrophoresis, the gels were analyzed with the Odyssey Infrared Imaging System (LI-COR).

For competition analysis, unlabeled double-stranded DNA that was identical in base sequence to the labeled probe was added in 5- to 20-fold molar excess relative to the labeled probe; this mixture was incubated at room temperature for 30 min before incubation with the labeled probe. For the supershift analysis, anti-RARα antibody (clone 9α-9A6; Active Motif) was added to the preincubated nuclear extract before addition of the labeled probe, and incubated at room temperature for 1 h; the final mixture containing probe was incubated at room temperature for 30 min.

### Chromatin immunoprecipitation (ChIP) assay

ChIP assays were used to study the interaction between RARα and *Csn3* promoter DNA in cells. P19 cells were treated with 1 µM of ATRA. After 3 h of this treatment, cells were treated with a crosslinking agent, 1% formaldehyde, and then disrupted via resuspension in SDS lysis buffer and sonication; this procedure yielded DNA fragments of ∼400 bp. Supernatants were incubated with anti-RARα monoclonal antibody (clone 9α-9A6; Active Motif) or control (non-specific) mouse immunoglobulin (IgG) (Santa Cruz Biotechnology) overnight at 4°C with rotation. The DNA-protein immunocomplexes were first precipitated with Protein G Sepharose 4 Fast Flow (GE Healthcare) and then eluted from the Sepharose. Eluate was treated with RNase A and proteinase K. To detect co-immunoprecipitated DNA, the immunoprecipitates were analyzed using PCR. The primer sequences and the expected size of the amplified fragment of each PCR were as follows: *Csn3* DR5 RARE ChIP (the region corresponding to −206/+1 of *Csn3* promoter including the putative DR5 RARE), 5′-CCTGCATGCTTATGACTCACA-3′ and 5′-CTCTTAGCTGCAGAGAAGACC-3′, amplifying a 207-bp product; negative control ChIP (a region distal to the target site, −739/−575 of 5′-region of *Csn3*), 5′-GTTTTGCAATCCATGCGCTAA-3′ and 5′-TTGCTTCAGCGTGTATTCCTC-3′, amplifying a 165-bp product. The PCRs were performed using AmpliTaq Gold 360 Master Mix (Applied Biosystems). PCR cycles were as follows: step 1 (initial denaturation), 95°C for 10 min; step 2 (amplification), 95°C for 30 sec, 60°C (for target region) or 62°C (for negative control) for 30 sec, and 72°C for 1 min; and step 3 (final extension), 72°C for 7 min. The non-immunoprecipitated supernatants (Input) were used as positive controls. The PCR products were analyzed on a 2% agarose gel. The gels were stained with ethidium bromide and visualized by UV illumination.

### Statistical analyses

Statistical analyses were performed using Microsoft Excel. The significance of the difference between groups was determined using the Student’s *t*-test. The values marked by an asterisk (*) differ with statistical significance (*P*<0.01) from indicated control values.
